# An overview of forensic operations performed following the terrorist attacks on November 13, 2015, in Paris

**DOI:** 10.1080/20961790.2020.1811487

**Published:** 2020-11-02

**Authors:** Antoine Tracqui, Céline Deguette, Tania Delabarde, Yann Delannoy, Isabelle Plu, Isabelle Sec, Lilia Hamza, Marc Taccoen, Bertrand Ludes

**Affiliations:** a Medico-Legal Institute of Besançon, Saint-Jacques Hospital, Besançon, France; b Medico-Legal Institute of Paris, Paris, France; c Forensic Medical Emergency Service, Hôtel-Dieu Hospital, Paris, France; d Université de Paris, BABEL, CNRS, Paris, France; e Medico-Legal Institute of Lille, Lille, France; f Sorbonne University, Paris, France; g Service d'Accueil des Urgences, Hôpital Avicenne, Bobigny, France

**Keywords:** Forensic sciences, terrorist attacks, Paris, forensic identification, firearms, explosives

## Abstract

On the evening of November 13, 2015, the city of Paris and its surroundings was hit by a series of attacks committed by terrorist groups, using firearms and explosives. The final toll was 140 people deceased (130 victims and 10 terrorists or their relatives) and more than 413 injured, making these attacks the worst mass killings ever recorded in Paris in peacetime. This article presents the forensic operations carried out at the Medicolegal Institute of Paris (MLIP) following these attacks. A total of 68 autopsies of bodies or body fragments and 83 external examinations were performed within 7 days, and the overall forensic operations (including formal identification of the latest victims) were completed 10 days after the attacks. Over this period, 156 body presentations (some bodies were presented several times) were provided to families or relatives. Regarding the 130 civilian casualties, 129 died from firearm wounds and one died from blast injuries after an explosion. Of the 10 terrorists or their relatives who were killed, eight died from suicide bombing, one was shot by police and one died from crush injuries due to partial collapse of a building following the police raid against a terrorist’s hideout after the attacks. All mass shootings were perpetrated with AK-47 or Zastava M70 assault rifles using 7.62 mm × 39 mm cartridges. In the case of ballistic injuries, death was most often obviously caused by craniocerebral injuries, extensive organ lacerations and/or massive haemorrhage. Among the terrorists killed by bombing, the lesion patterns were body transection, multiple amputations, extreme organ lacerations and the presence of foreign bodies owing to the shrapnel load (steel nuts, glass fragments) or the explosive charge fastening system of the devices. This discussion highlights the particular difficulties of interpretation encountered within the framework of ballistic injuries, a conclusion that should lead to a modest and realistic approach in these exceptional situations where forensic operations involve a very large number of victims in a constrained time.

## Introduction

**Figure 1.**
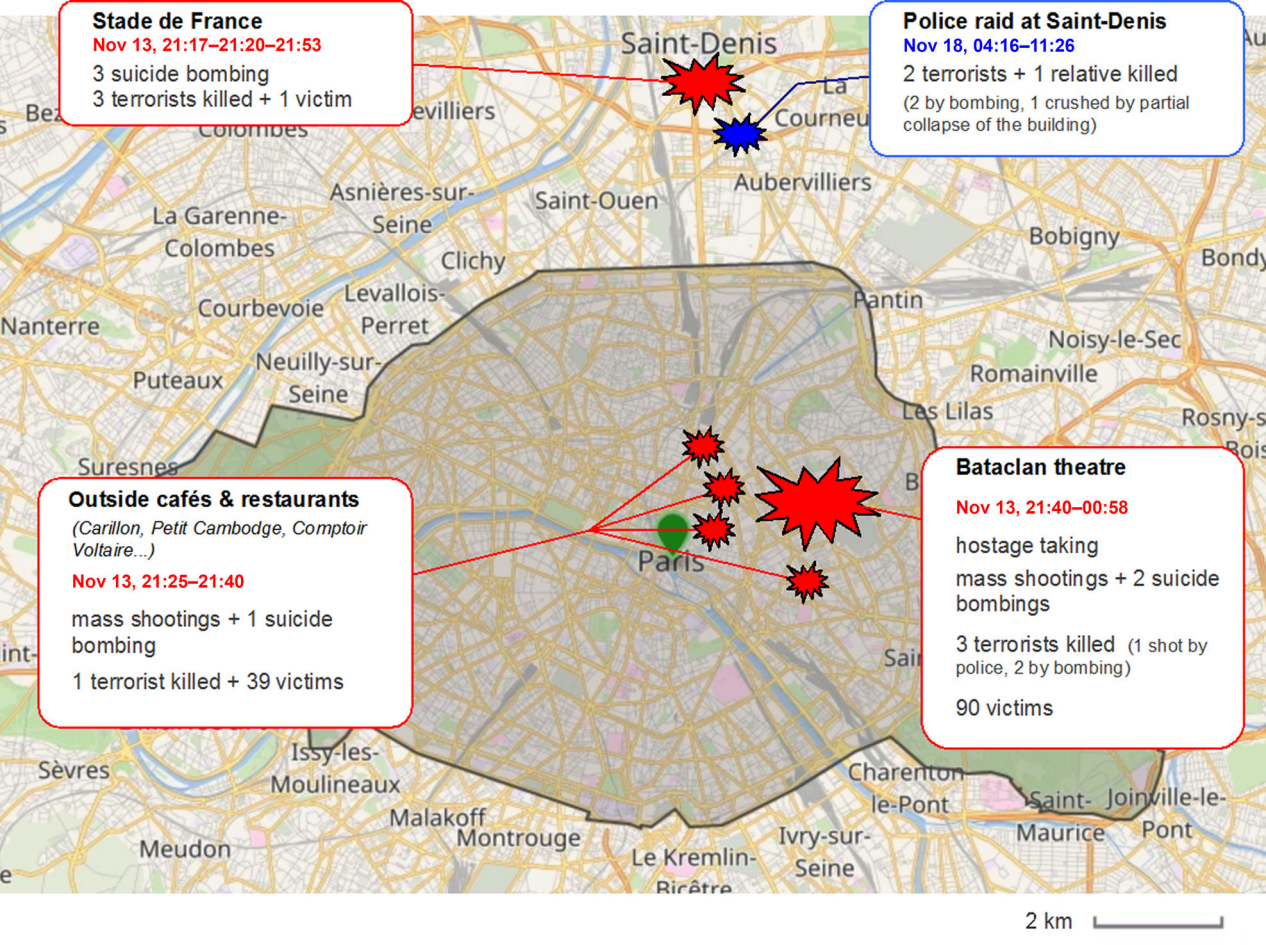
Chronology and geographical distribution of the terrorist attacks and subsequent events in Paris from November 13 to 18, 2015.

On the evening of November 13, 2015, the city of Paris and its surroundings was hit by a series of coordinated attacks committed by terrorist groups operating in three different locations (Figure 1). The first attacks, involving three successive suicide bombings, occurred in the vicinity of the Stade de France in Saint-Denis, a suburban city located north of Paris. The first attacks were followed by a series of mass shootings on the terraces of cafés and restaurants, then by a hostage taking inside the Bataclan, a theatre located in the 11th arrondissement of Paris, where the highest number of victims was sustained. Five days later, on November 18, 2015, a police raid conducted in Saint-Denis resulted in the death of two terrorist survivors of the previous attacks, and a female relative of one of the terrorists. The final toll was 140 people deceased (130 victims, nine terrorists and one relative of a terrorist) and more than 413 injured, making these attacks the worst mass killings ever recorded in Paris in peacetime.

Several articles [1–4] have already been written on the organisation of medical care for people injured during these types of criminal actions, but to our knowledge, no article has yet addressed the overall medicolegal issues. The purpose of this article was to present the general scheme and course of the forensic operations carried out as a result of these attacks, with particular emphasis on the difficulties of interpretation encountered within the framework of ballistic injuries.

## Materials and methods

### Timeline and strategy of forensic operations

During the day of November 14, 2015, a total of 123 bodies and 129 body fragments, among which 41 corpses were labelled “X” (unidentified and without putative identification) or “X supposed to be” (unidentified with putative identification), were transported to the Medicolegal Institute of Paris (MLIP). Over the following days until November 19, the bodies of victims who survived the attacks but later died in the intensive care units (ICUs) of various Parisian hospitals were also dispatched to the MLIP. Victim triage and imaging operations began in the afternoon of November 14, and the autopsy operations began the next morning.

The strategy of forensic operations was decided in consultation with the public authorities (Prosecutor of Paris), and were aimed at reconciling the following imperatives: identifying all victims, determining the causes of death, reception and accompaniment of victims’ families and quick return of bodies to the families. Of the three options available (indiscriminate autopsy of all bodies, external examination + DNA sampling for all bodies and targeted autopsies), the first option was rejected because of a foreseeable duration that was considered excessive (estimated time required: 12–15 days), and the second option was rejected for fear of collecting insufficient evidence. The final decision was to perform targeted autopsies, restricted to the following cases: a) terrorists’ bodies; b) “X” or “X supposed to be” bodies; c) cause of death uncertain at external examination; d) victims of the Bataclan site with intracorporeal ballistic material at imaging (because of the police raid carried out to stop the hostage taking, it was necessary for judicial reasons to formally identify the nature of the weapons responsible for the fatalities), and e) injured people who died in the ICUs. Bodies that were not designated for autopsy underwent thorough external examination (EE) completed by imaging and DNA sampling.

### Victim identification

The identification process was conducted by the Disaster Victim Identification (DVI) unit of the French National Police under the authority of the Paris public prosecutor’s office. Nevertheless, first at the scene were criminal police units who recovered the bodies, and a great number of identifications were made at the scene on the basis of personal effects and facial recognition, which introduced initial identification uncertainty. It was subsequently decided to review all of the identifications made at the scene during the initial DVI procedure while other identifications were made in accordance with the complete DVI protocol [5]. A postmortem coordination (PMC) centre located at the MLIP was responsible for gathering primary (DNA profiles, fingerprints and dental records) and secondary (e.g. unique and identifiable scars, tattoos, clothing, jewellery and signs of known medical disease) identifiers collected during the examination of the corpses at the attack sites, and during the autopsies/EEs and other forensic operations. Meanwhile, antemortem coordination (AMC) centres organised outside the MLIP at the host sites for families (e.g. Ecole Militaire) were tasked with collecting data from the relatives or medical practitioners caring for the potential victims. The reconciliation coordination (RC) commission located at the MLIP was responsible for cross-referencing the data from the PMC and AMCs, and pronouncing provisional or confirmed identifications. Once identification was confirmed, a magistrate from the public prosecutor’s office completed the judicial burial form.

### Autopsies and EE procedures

A team of 15 experienced pathologists (12 from the MLIP staff and three from the Criminal Investigation Institute of the French National Gendarmerie, CIING) was formed and divided among four autopsy tables, of which three were devoted to autopsies and one to EEs. A fifth autopsy table was reserved for the MLIP's routine autopsies, which continued to be performed on a day-to-day basis. Additional staff consisted of seven radiologists, four odontologists specialised in postmortem identification, one forensic anthropologist and four specialists in ballistics and explosives from the CIING.

Quick triage was performed at the entrance to the MLIP by two pathologist coordinators for each body and body fragment, with preliminary imaging, to help choose between subsequent autopsy or EE. Standard projection X-ray examinations were performed in the imaging room of the MLIP, whereas CT scans were obtained at two neighbouring hospitals (Hôtel-Dieu and Sainte-Anne).

Each autopsy was performed by three pathologists assisted by at least two investigators, with one investigator specialised in forensic identification and one in ballistics/explosives. After consulting the imaging material, the successive steps were as follows: a) undressing the bodies and describing the deceased’s clothes and personal effects; b) fingerprinting followed by the first stage of the EE, which was mainly devoted to collecting identifying elements, in accordance with the International Criminal Police Organization (INTERPOL) DVI guide; c) the second stage of the EE, which mainly focused on collecting lesional elements (foreign bodies); d) complete dissection of the body, including collecting samples for toxicology, anatomic pathology and genetic analysis, and e) carefully collecting all foreign bodies and elements of ballistic origin, or those likely to have come from explosive devices. Steps a), b) and c) were performed in the same way for bodies designated for EEs.

### Reception and care of the victims’ relatives

Primary psychological support for relatives was ensured as soon as November 14 by members of a medico-psychological emergency unit housed in temporary premises in close proximity to the MLIP, whereas information on formalities and funeral procedures was provided by the administrative staff of the MLIP. Family viewing of the deceased was systematically carried out by a clinical psychologist from the MLIP.

## Results and discussion

Thanks to the procedures detailed above, a total of 68 autopsies of bodies or body fragments (in all cases with prior EE) and 83 EEs (without subsequent autopsy) could be performed within 7 days, with the last autopsies completed during the afternoon of November 20. The overall forensic operations (including formal identification of the latest victims) were completed on November 23. Over this period, 156 body presentations to families or relatives were provided in the funeral chambers of the MLIP (some bodies were presented several times, if requested by families).

Of the 140 people deceased during or following the attacks, all 130 civilian casualties died from firearm projectile wounds except one who died from blast injuries following one of the explosions at the Stade de France. Among the nine terrorists killed, seven died from suicide bombing, one showed a combined mechanism of death (explosion of explosive belt and shooting) and one was shot by police during the assault on the Bataclan hostage-taking site. A female relative of one of the terrorists died from crush injuries due to partial collapse of the building following the police raid against the terrorists’ hideout in Saint-Denis.

As shown in the police inquiry, all mass shootings were perpetrated using AK-47 or Zastava M70 assault rifles, which are quite similar in design. Both weapons use widely available 7.62 mm × 39 mm cartridges [6]. The projectile in these cartridges is a full metal jacket (FMJ) bullet, with a muzzle velocity range of 715–730 m/s. Complete bullets, intact or slightly deformed, were present in less than one-third of the bodies. In many cases, they were found superficially embedded in the subcutanous or muscular soft tissues without prior bone impact, suggesting that the bullets had already lost most of their kinetic energy before penetrating the victim (perhaps by previously passing through other people’s bodies).

**Figure 2.**
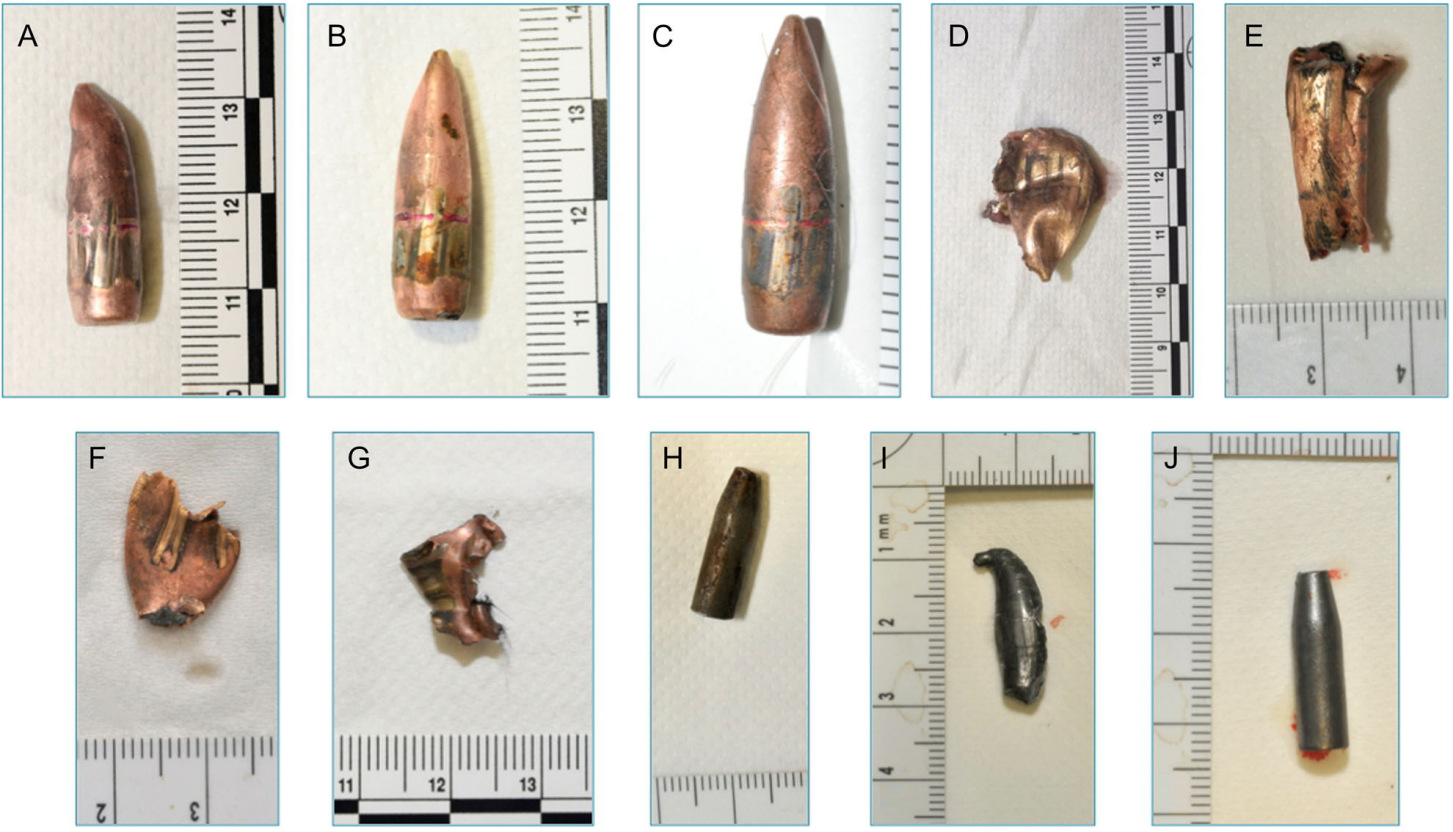
Examples of ballistic material collected from victims' bodies. (A)–(C) Intact or slightly deformed 7.62-mm bullets. (D)–(G)  Jacket fragments. (H)–(J) Steel cores.

**Figure 3.**
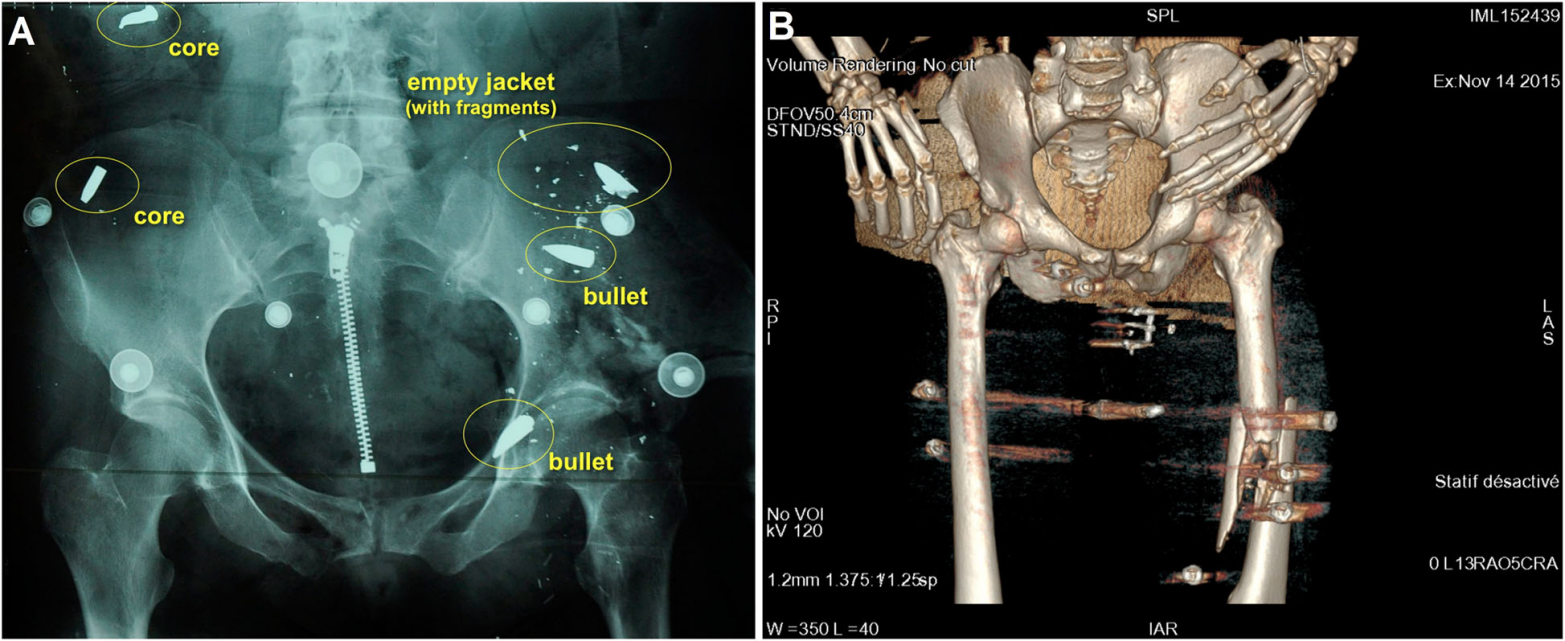
(A) Representative standard pre-autopsy X-ray image allowing identification of various kinds of intracorporeal ballistic material. (B) Representative 3D-reconstructed pre-autopsy CT imaging lacking precision for identifying the various kinds of intracorporeal ballistic material.

More commonly than complete bullets was the presence of jacket fragments, together with complete or split steel cores, a pattern that was found repeatedly from one corpse to another (Figure 2). These findings suggested a high degree of projectile fragmentation, which was not expected *a priori* with the type of FMJ bullets used in this attack. A possible explanation is that, especially in the Bataclan theatre, the shots occurred in a dense crowd gathered in a very confined environment, leading to multiple ricochets and successive impacts. Unexpectedly, standard X-ray images proved to be much more useful in identifying the type of ballistic material present in the bodies prior to autopsy compared with CT images (Figures 3). This was mainly due to metal streak artifacts (e.g. by beam hardening phenomena), making the shape of the foreign bodies difficult to appreciate in CT images. Additionally, standard radiographs were performed at the MLIP while CT scans were obtained in hospitals far from the MLIP. The images used in the autopsy rooms were mainly in volume rendering technique (VRT) format (Figure 3B), which was more difficult to interpret ballistically compared with MIP format.

Regarding wound ballistics, causes of death were most often obvious by craniocerebral injuries, massive haemorrhage and/or extensive organ lacerations, as is usually described with high-energy military rifle bullets [7]. Despite the participation of experienced specialists in ballistics throughout the autopsies, gunshot characteristics were much harder to assess in many cases because of the following: a) multiple skin perforations (from 1 to 32 per victim) and b) atypical wound features (due to high-energy projectiles, pre- or post-entry destabilisation or fragmentation), which made it difficult to distinguish between entries and exits. Shooting distances were almost always impossible to estimate, especially because of the interposition of clothing. Even the number of shots per victim remained uncertain in approximately one third of cases due to the high frequency of re-entry wounds (e.g. limb-to-limb, limb-to-torso or upper limb-to-head), and the often-recorded presence of projectiles in the clothing or body bags without it being possible to say whether the projectiles actually penetrated the victim, or if their presence was only fortuitous.

Of the eight terrorists deceased from suicide bombing, seven were killed by operating their own explosive belt and one died from blast lesions of extreme severity due to being in close proximity to one of his accomplices at the moment the accomplice triggered his explosive device. Subsequent analyses showed that the explosive used was acetone peroxide, which is easy to produce and has been widely used by terrorist groups in many attacks since 2001. The pattern of the lesions was very repetitive and involved body transection, multiple amputations, extreme organ lacerations and the presence of foreign bodies owing to the shrapnel load (steel nuts, glass fragments) from the devices. Another specific feature was the presence in all bodies of fragments of blue adhesive tape, likely originating from the explosive charge fastening system. Specific patterns of bone trauma providing a quick distinction between victims and terrorists to assist the medicolegal identification process have been presented and discussed elsewhere [8].

## Conclusion

Thanks to the logistics implemented following the attacks of November 13, 2015, in Paris, all the medicolegal operations performed on the 140 people who died (130 victims, nine terrorists and one terrorist’s relative) could be performed within 7 days, and conclusive identification of the last bodies was completed on November 23, 10 days after the perpetration of the criminal actions.

This unprecedented event in France with regard to the number of victims and the complexity of forensic operations once again demonstrated the imperative need for a multidisciplinary approach involving experienced pathologists, anthropologists, odontologists and radiologists working in close collaboration with dedicated law enforcement units, and in particular, specialists in the field of ballistics and explosives [9–11].

Despite this optimal concentration of means and skills, a key lesson learned from this experience is that many cases remained incompletely solved on a ballistics level, i.e. projectile trajectories, shooting distances or even the number of gunshots per victim. Our conclusion is that when dealing with a very large number of victims in a constrained time while facing multiple intricate imperatives, not just forensic imperatives, the expectations of the medicolegal team should remain modest and realistic. Formal identification of all victims, determining the causes of death and recovering evidence material are mandatory targets, but a precise determination of the circumstances of each fatality, as should be the case in individual criminal situations, does not necessarily have to be considered an attainable or even desirable goal.
